# Warm and Hot Water in Surgery

**Published:** 1875-10

**Authors:** C. Henri Leonard

**Affiliations:** Detroit, Mich.


### "WARM AND HOT WATER IN SURGERY."

BY C. HENRI LEONARD, M. D., DETROIT, MICH.

This article is not written so much to call attention to the efficacy of this plan of treatment, as to set aright some of the current opinions as to its originator.

The recent publication of Prof. Hamilton's *brochure* in your columns has more particularly called my attention to the matter. Therein it seems to be fathered upon the French surgeon, Amussat; possibly, though, in the translation Prof. Hamilton refers to, *The Use of Water in Surgery*, proper reference to old authorities has been made. I have not the *Buffalo Journal* at hand to refer to, in which Prof. Hamilton's translation was published, so presume upon your patience to the following amount of quotations from the good old Hippocrates.

Hippocrates was a firm believer in the efficacy of hot and warm water; indeed, his works upon "Ulcers," "Fractures" and "Dislocations" teem with it. In his 22d Aphorism, sec. V., (Adams' translation), he says: "Heat is suppurative, but not in all kinds of sores; but when it is, it furnishes the greatest test of their being free from danger. It softens the skin, makes it thin, removes pain, soothes rigors, convulsions and tetanus. *It is particularly efficacious in fractures of the bones, especially of those which have been exposed, and most especially in wounds of the head, and in mortifications;* in herpes exedens, of the anus, of the privy parts, the womb, the bladder, in all these cases heat is agreeable, and brings matters to a crisis, but cold is prejudicial and does mischief."

The italics, etc., in the above are my own. You notice it covers almost entirely the ground gone over by Prof. Hamilton, and is laid down in so positive a way, as really to surprise me that all reference to this statement by Hippocrates should have been overlooked. There is another point to be taken into consideration—"the womb." Dr. Emmet lays claim to the originality of hot-water uterine applications; he has told me so by word of mouth, adding that he got the idea from the late Dr. Pitcher, of this city. On p. 21 of his brochure upon "The Philosophy of Uterine Disease," published last year by the Appletons, he refers to the same in a foot-note, ending it with: "Certainly no one in this country is on record as an advocate for the practice previous to myself; and, as far as I have been able to ascertain, the same is true in regard to the practice of gynæcologists abroad." Yet, you can see how plainly the practice (in theory at least) was understood by the old Hippocrates. I shall refer to this hot-water uterine treatment, in another Hippocratic statement, before I am through.

It was customary with Hippocrates, as the aphorism indicates, to use hot water freely in fractures, etc.; hence it is with no surprise that we find the following in his "Surgery:" "As to the temperature and quantity of the water used, its heat should be just such as the hand can bear (about 110° Fahrenheit, C. H. L.), and it ought to be known that a large quantity is best for producing relaxation and attenuation (does he not mean by that what we now call a contraction of the arterioles, veinlets and capillaries, *a la* Emmet? C. H. L.) whereas a moderate quantity is best for incarnating and softening. The limit to the effusion is, to stop when the parts become swelled up, and *before the swelling subsides* (the stage of 'pelvic contraction,' of Emmet, C. H. L.); for the parts swell up at first, and fall afterwards."

In "Fractures" he says: "When you remove the bandages, you must pour hot water on the arm and bind it up again." In another place, in the same treatise, he says: "At each time the bandages are taken off, much hot water is to be used; for *in all injuries at joints* the affusion of hot water in *large* quantities is to be had recourse to." In still another place in the same treatise, he refers to the practice in this wise: "We must avoid wetting it (an exfoliating fracture) at the beginning with anything *cold*."

In the "Mochlicus" he says: "They (dislocations) should have more copious affusions of water" (hot). He reiterates the same expression still farther on when treating of the special dislocation of the os calcis.

Again, in the "On the Use of Liquids," one of the treatises generally ascribed to Hippocrates, we have this hot-water question pretty thoroughly canvassed. I give but three condensations: Hot water promotes ulceration in all cases, softens the skin, attenuates it, is anodyne, soothes rigors, spasms and tetanus, etc. Again: It is most particularly applicable in fractures, when the bone is laid bare, and especially in injuries of the head. [I have found the most immediate relief, and really quite permanent, from hot-water douches (two quarts or more) in cases of acute nasal catarrh. In my own person, I rarely employ anything else. I always add a tablespoonful of common salt to the water, and when taken at or near bedtime, it produces speedy languor and sleepiness; probably from the contraction of the brain arterioles. Some of the most violent cases of hemicrania (paralytica, probably,) I have found greatly relieved by hot fomentations, when ice was unbearable; I remember a case, in particular, where everything had been used most assiduously for twenty-four hours, and yet the patient, a male, about forty years of age, was fairly blind with pain. At last hot water suggested itself, and after the second removal of the flannel compress, the patient was in a sound sleep. He had no return for some ten days, the longest time of immunity he had had in some time.] Again, quoting from this treatise: Hot water agrees with all ulcerations in herpes exedens, in blackened parts, (gangrenous?) and in diseases of the ears, anus and womb.

The quotations certainly show that hot water was in common use by the ancients for the very same purposes that we now apply it; the only difference seeming to be in the manner of its employment in the case of fractures; they employed prolonged affusions, whereas now we are recommended by Hamilton to use the constant bath, or else, (still nearer the Hippocratic manner,) "thoroughly saturated fomentations."

Since I received verbally from Dr. Emmet the wonderful effects of hot vaginal douches, I have had them in constant use among my female patients. They are one of the best "soporifics" that a hysterically nervous female can take. I always take especial pains to order one at bed-time, in these cases, and rarely fail of getting a good night's repose for my patients. They soon "see how it is themselves," and become actually solicitous for their nightly douche. Yet, even this soporific tendency was well known in the days of Hippocrates, for he speaks of it as one of the *sequelœ* of hot water applications.

Prof. Agnew, of New York, was the first, and, if I remember aright, the only one I have heard to recommend continuous baths of hot water for the eyes, in certain corneal irritations, and chronic granulations. Following his advice, I have found good results to follow such treatment. In this Hippocratic treatise I have just been quoting, I find the following: "In pains, suppurations, pungent tears, and deep ulcers of the eyes, hot water is most expedient; when the eyes are merely red, and free from pain, cold is to be preferred." The sentiments here correspond almost exactly with those I heard from Prof. Agnew's lips.

I wish to quote one of the Hippocratic thoughts upon cataplasma before closing, for it covers ground that has been, apparently, lost sight of in these later years. I have italicised the special thought to which I wish to call attention. The passage is found in Hippocrates' treatise upon "Ulcers," and reads: "When you seem to require a cataplasm, it is not the ulcer alone to which you must apply it, but *to the surrounding parts*, so that the pus may escape and the *hardened parts become soft.*"

I do not wish to rake up too much "dead" literature; still I cannot help thinking that a judicious acquaintance with these old authors will add much to modern Æsculapian enlightenment. *Chicago Medical Journal and Examiner.*

